# Dying like a dog: the convergence of concepts of a good death in human and veterinary medicine

**DOI:** 10.1007/s11019-021-10050-3

**Published:** 2021-09-15

**Authors:** Felicitas Selter, Kirsten Persson, Johanna Risse, Peter Kunzmann, Gerald Neitzke

**Affiliations:** 1 Institute for Ethics, History and Philosophy of Medicine, Hannover Medical School, Hanover, Germany; 2 Institute for Animal Hygiene, Animal Welfare and Farm Animal Behaviour, University of Veterinary Medicine Hannover, Hanover, Germany

**Keywords:** Death and dying, End-of-life decisions, Euthanasia, Palliative care, Veterinary ethics

## Abstract

Standard views of good death in human and veterinary medicine considerably differ from one another. Whereas the good death ideal in palliative medicine emphasizes the positive aspects of non-induced dying, veterinarians typically promote a quick and painless killing with the aim to end suffering. Recent developments suggest a convergence of both professions and professional attitudes, however. Palliative physicians are confronted with patients wishing to be ‘put to sleep’, while veterinarians have begun to integrate principles and practices from hospice care. We will argue that the discourses on good human and animal deaths are not distinct, but that they interact and influence each other. On the one hand, veterinary medicine adapts techniques like chemotherapy or sedation from palliative end-of-life care. On the other hand, philosophers, veterinarians, pet owners, patients and the general public alike make certain assumptions about the (dis)analogy of human and animal dying or killing. Unfortunately, these interactions have only scarcely been reflected normatively, especially on the part of human medicine. Conflicts and misattributions with potential serious negative consequences for the (animal and human) patients’ wellbeing are provoked. For these reasons, palliative physicians and veterinarians are invited to engage in the debate around human and animal end-of-life care.

## Introduction

In many Western countries, most humans and companion animals^1^ today die in medical institutions and nursing homes (Klinkhammer 2012; Ohr 2019). Death is often preceded by an intentional decision against the prolongation of life, by either actively inducing death or letting the patient die. Human and veterinary medicine also have standard views of how, when and where dying should ideally take place. In veterinary medicine, euthanasia represents the most common procedure to end or prevent suffering, whereas it is typically not an option for human patients.

In medical ethics, the idea that there are objective criteria that define death as either good (and therefore acceptable or even aspired) or bad (and therefore to be rejected and avoided) received much criticism in the past for pushing individual preferences and values into the background. Accordingly, the quality of death and dying is now commonly defined as “the degree to which a person’s preferences for dying and the moment of death agree with observations of how the person actually died, as reported by others” (Patrick et al. 2001, p. 721). Studies have shown that a multitude of criteria affects concepts of a good death, e.g., the respondent’s age, health, prognosis, finances, culture, spirituality and even personal experience in terminal care (Hutter et al. 2015, p. 1299). This implies that there is no universal definition of what exactly makes a death ‘good’ (Meier et al. 2016).

Moreover, religious rites and conventions are continuing to lose meaning in secular societies. Instead, several modern *dying scripts* or *ideals* have emerged, providing guidance and inspiration instead (Green 2008; Streeck 2019). These ideals often share important elements, e.g., the absence of pain and distress, timeliness, being in control, predictability and a certain preparedness, but can also considerably differ from one another, especially with regard to the question whether death should be actively induced.

Concepts of a good death have only very recently found access into veterinary medicine. For a long time, a good death was (implicitly) understood to be equivalent to the humane and painless killing of animals; the question not so much being *whether* but rather *when* euthanasia would be the right thing to do (Leary et al. 2020; FVE 2019). With the rise of palliative veterinary care and the animal hospice movement, alternative views emerged.

It will become evident that current trends hint towards opposite developments in human and veterinary medicine, indicating a convergence of the concepts of good human and companion animal deaths, respectively. We will argue that these considerations do not constitute two completely distinct discourses, but interact.^2^ The inferences between the two professions have only scarcely been reflected normatively, especially on the part of human medicine, which provokes certain conflicts and misattributions with potential negative consequences for the patients’ wellbeing.

In order to get a hold of current views of good death and dying^3^ in human and veterinary medicine, it is essential to understand where they originated. We will provide brief, and thus necessarily simplified, overviews and afterwards show why these developments could be interpreted as a convergence.

## The good human death

Death had long been a communal event. Over time, however, it became increasingly private. During the 1960s, death was not something to be talked about and if, only “in a polite, euphemistic, hushed manner” (Green 2008, p. 3). Deceit of the terminally ill regarding their prognoses was thought to be a kindness, accentuated by families and health care providers alike. British anthropologist Geoffrey Gorer famously asserted that the absence of public mourning and the prudence surrounding the topic had generated a “pornography of death” (Gorer 1965 as cd. in Green 2008, p. 4). Death and the whole dying process, once perceived as a natural, inevitable part of life, had turned into something embarrassing that needed to be hidden.

At the same time, advances in medicine and technology not only led to an increase in life expectancies, but suddenly made it possible to keep terminally ill patients alive much longer than before, raising questions about quality of life and the appropriateness of certain therapeutic measures (Neitzke et al. 2019). Death was standardly viewed as the medical profession’s ‘enemy’ during this time. This attitude demanded of the health care professionals to do everything possible to keep a patient alive, regardless of their condition, prognosis and preferences (Green 2008, p. 2; Kübler-Ross 1975, p. xix ff).

Both the hospice movement and the right to die movement resolutely pushed back against this view. They believed that not death but a poor quality of life should be regarded as the enemy and that a ‘good death’ was possible (Hurst and Mauron 2006). Even though palliative care providers and right to die advocates have been argued to have more values in common than might be thought at first glance, they also considerably differ from one another with regard to their visions of a good death (ibid. 109). As we are concerned with these visions or ideals, we will put a disproportionally large emphasis on the differences rather than the common ground here (e.g., to alleviate suffering and to maintain self-determination). The following paragraphs are thus not to be misread as exhaustive depictions of palliative care and euthanasia positions. Instead they should be understood as attempts to put attention to and further elaborate on their distinct views on induced death.

### The palliative good death ideal

The original hospice movement redefined death as a natural part of life and rejected irrational attempts to either deny or defeat mortality. Instead, endeavors to accept and talk about death, both on the individual as well as the societal level, were actively encouraged. Dying was depicted as “the last great opportunity for ‘growth,’ a time of personal transformation, even triumph” (Green 2008, p. 3), providing “a chance to discover life’s true meaning” (Kübler-Ross 1975, back cover). Even intense suffering and existential fear could be positively reinterpreted as parts of this transformative process.

Even though Kübler-Ross stressed that her phase model was not to be read as a universal recipe, her work was later interpreted in exactly that way. It was inferred that palliative patients *necessarily* needed to travel through all five stages of grief (denial, anger, bargaining, depression and acceptance) in order to die well. The resulting rigid good death ideal has been criticized from different angles, most notably for implementing a socially and culturally powerful script ‘how to die properly’ and thus pressuring patients into “dying according to what the healthcare professionals consider to be a ‘good death’” (Cottrell and Duggleby 2016, p. 707).

On the one hand, modern palliative care has certainly outgrown this authoritative model by acknowledging the “multiplicity of beliefs and communication patterns evident in the collective of dying patients” (McNamara 2004, p. 936). Palliative care follows an entirely individual as well as holistic care approach, providing medical, emotional as well as spiritual support for all people involved in the dying process, e.g., the patients, their families and caregivers, tailored to their specific needs. Its focus lies on the enhancement of quality of life and on enabling patients to “live as actively as possible until death” (Sepúlveda et al. 2002, p. 94). On the other hand, a certain view of a good death, rooted in the original hospice movement, continues to be held. It will be called the *palliative good death ideal* here and hereafter, even though we wish to stress that it is, of course, neither the sole good death ideal within palliative care, nor shared universally by *all* palliative care providers in Europe.^4^ In our depiction, we will refer to Radbruch et al. (2016). It makes no explicit mention of the view we wish to describe, as the authors are not concerned with good death ideals underlying the palliative care philosophy in this article. Yet, they exemplify an influential critical attitude against euthanasia from a palliative care perspective by referring, among others, to the WHO-definition, according to which death should neither be hastened nor postponed. The article by Radbruch et al. (2016) thus has the purpose of drawing attention to rather than being taken as a reference for a certain position.

The ideal’s close proximity to the original hospice movement becomes particularly clear in its endeavors to dissociate from euthanasia^5^ movements. Two aspects are of particular importance for our purpose. First, palliative care is said to be life-affirming, because it strongly believes that “even in a patient’s most miserable moments, sensitive communication, based on trust and partnership, can improve the situation and change views that his or her life is worth living” (Radbruch et al. 2016, p. 114).^6^ This is at least compatible with the observation that “[p]alliative care providers […] sometimes seem to claim that patients who think that their life is irretrievably bad and ‘worth not living ‘ are always wrong. A patient may accurately judge their current quality of life to be unacceptable, but adequate care would always increase their quality of life to the point where they would reconsider” (Hurst and Mauron 2006, p. 108).

The second aspect concerns the palliative good death ideal’s emphasis on ‘natural’, non-induced death. Through its efforts to retain the life-affirmative character of medicine but simultaneously regard dying “as a normal process”, “neither to [be] hasten[ed] or postpone[d]” (Sepúlveda et al. 2002, p. 94), good dying from a palliative care perspective also requires modesty, both from the health care team and the patient.^7^ The health care team must not unnecessarily interfere with the dying process by intentionally prolonging or shortening it, and the patient is to submit to the end of life by neither resigning too early nor desperately clinging to life too long. This implies a right point in time for death to occur, that should not (or cannot) be externally determined, neither by the physician nor, importantly, by the patient herself. It is crucial to recognize that this prima facie applies to all patients, therefore, irrespective of their individual views of a good death or their status as (in)competent decision-makers, (non-)autonomous beings etc. There must be something, from this point of view, which makes non-induced death preferable *in principle* to an induced death, therefore.

One way to do so is to hold onto the view of dying’s transformative potential. The position of death’s transformative potential traces back to the beginnings of the hospice movement (and beyond) and is advocated today in modern variants. Steffen-Bürgi even goes as far as to state that it is the ultimate goal of palliative care to assist in perceiving the possibilities for personal development and inner maturation (Steffen-Bürgi 2009, p. 274). Egan City & Labyack, in describing hospice palliative care for the twenty-first century, express their strong belief that dying represents opportunities for growth and development, while emphasizing that the question how exactly this time is to be designed is up to the patients and their families. They write:

Although the last phase of life and relationship is a difficult time, with sensitive support it can be a time of tremendous growth and opportunity for the person who is dying and for loved ones – such as in the finding of meaning and purpose in one’s suffering, the value of one’s life accomplishments, the deepening of relationships, and the personal spiritual significance of the experience. (Egan City and Labyak 2010, p. 57f).^8^

Swiss palliative care providers in a recently conducted qualitative interview study expressed their belief that death can be an opportunity for personal growth, following Elisabeth Kübler-Ross (Mezger 2018; see also Lüddeckens et al. 2016 for a report of the project).

Zimmermann et al. (2019) accordingly concludes that one understanding of death within today’s palliative care regards death as an inherent part of life, which is usually linked to a rejection of euthanasia among the respondents. Zimmermann emphasizes that religious convictions and a certain understanding of dignity associated with them play a decisive role (Zimmermann et al. 2019, p. 180). There are also more recent interpretations of a transformative good death ideal that incorporate ideas from existential philosophy and psychology (Renz et al. 2013; Schnell 2019). Accordingly, some authors prefer to speak of alternative religious beliefs because the ideas and practices associated with them do not stand in a narrower framework of traditional faith communities and traditions (Lüddeckens et al. 2016, p. 2). The research group around these authors asserts in their project report that alternative religiosity is found among patients themselves, but also among palliative care providers. Particularly relevant, according to the authors, is the concept of the ideal of personal development toward a higher self. This also shows in a critical to negative attitude toward palliative sedation (Lüddeckens et al. 2016, p. 4f).

It remains speculative how widespread this position is among European palliative care providers, but it is neither a negligible position nor is it exclusively attributable to personal rather than professional attitudes. A palliative nurse might strongly believe that a patient would die much ‘better’ if he shared a certain view of growth on death and dying and even argue for a change of society’s views on these matters, while still respecting the patient’s values and do everything to fulfill his wishes, in line with the palliative care approach. This is no contradiction.

### The euthanasia good death ideal

Similar to Saunders and Kübler-Ross, the pioneers of the *euthanasia* or *right to die movement* took offense at an overly technical, paternalistic medical system and strived to ensure a dignified death. With regard to how death should ideally occur, they came to very different conclusions, however.

The palliative good death ideal acts on the assumption that a patient’s dignity and quality of life can be preserved right until death. Helping dispirited patients in recognizing the value of their remaining lives has been described as one of the main goals of palliative care. Right to die organizations, in contrast, regard a patient’s own judgment ceteris paribus as *will* that must be taken seriously. The individual alone defines at which point her life has or will become meaningless or unbearable and when the time is ripe to die a good, still dignified death. According to this ideal, self-determination is extended from the circumstances of the dying process to the time of death itself. The World Federation of Right to Die Societies believes accordingly that “all those who fully appreciate the consequences of carrying out their wish to die and who take into account the reasonable interests of others should have access to a peaceful death at the time of their choice” (WFRtDS 2019).

It is crucial to recognize that a voluntary (de)termination of death is not applicable to all patients, but only to those with the ability to make informed and autonomous choices in the first place. A shortening of the dying process would be regarded as beneficial to many other patients as well, from the perspective of at least some euthanasia advocates. The Netherlands, for instance, do not only acknowledge voluntary euthanasia requests but also practice non-voluntary euthanasia in pediatrics.

In light of this, it may be argued that a ‘natural’, non-induced death could become undesirable for many, especially—but not exclusively—for non-religious or non-spiritual people. Actively inviting death can be interpreted as a denial of the dying process. Cottrell and Duggleby (2016) even go as far as to claim that this preference can be observed on a societal level:

The dying process seems to have been rendered insignificant and has therefore been discarded. Though death itself is openly discussed, the dying process is so negatively characterized or perceived that it is now avoided entirely. Contemporary Western society, once a death-denying society, is now increasingly a death-accepting, but a dying-denying, society. (Cottrell & Duggleby 2016, p. 710)

In concert with this suggestion, the acceptance of euthanasia and PAS has continued to increase since the 1980s and is currently high among the general public in Western European countries (Cohen et al. 2013). Corresponding laws have been passed since then in European countries, Australia and North America. This trend is commonly interpreted as a sign that “euthanasia will eventually become legal” (Cohen et al. 2013, p. 380) in a growing number of Western countries (but see Radbruch et al. 2016 for an alternative reading).

## The good companion animal death

During the twentieth and twenty-first century, several scientific and social developments led to an increasing ascription of moral status to animals. The cognitive turn sparked a new interest in animals’ mental and emotional inner world, while animal ethics simultaneously gained academic significance. On the societal level, the emergence of one- and two-person households transformed the relationships between animals and their owners^9^ because singles, couples without children and elderly people living alone often form particularly close bonds with their pets, similar to the relation between a parent and a child or between life partners (Joswig 2014, p. 17ff; Rollin 2011, p. 653f). A pet’s death can occasionally result in grief as intense as after the loss of a close family member (Bishop et al. 2016, p. 349; Schmitt 2019).

In correlation with the increased moral status of pets and a higher social willingness to spend more money on their medical care, there is a growing demand for high-quality veterinary services. Accordingly, the profession has undergone major transformations, especially in small animal practice. Several specialties have evolved, such as oncology or geriatrics. In the US, hospice care and palliative medicine are currently under consideration as a future specialty (Bishop et al. 2016, p. 351).

The advances regarding veterinary diagnostics and therapy entail that even in the event of an unfavorable prognosis, there may be alternatives to immediate euthanasia. This requires a fundamental decision by the patient’s caregiver, similar to the one in human medicine, i.e., whether further treatment should follow a more aggressive strategy, focusing its efforts on the prolongation of the patient’s life, or a palliative route, preserving the patient’s best possible quality of life. The two strategies are not mutually exclusive, of course, as even an aggressive care-treatment plan must always assess and respect quality of life, but they represent two nonetheless very different routes (Bishop et al. 2016, p. 347).

Even in case of a non-curable cancer diagnosis and provided that veterinarian and owner have genuine interests in choosing what is best for the patient regardless of financial or other costs, several options may be available, therefore: from instant euthanasia at the veterinarian’s practice over palliative treatment until the animal’s ‘natural’ death up to the aggressive treatment of the tumor at an oncology clinic with the purpose of maximum life-prolongation. Thus, notions of what a good animal death actually entails become more and more relevant.

Unfortunately, veterinary ethical literature on the concept of good death is scarce. Furthermore, the multitude of aspects shaping the ethical discourse of animal death and the morality of killing are not apprehended in veterinary medicine, nor do veterinarians seem to be aware of the many philosophical assumptions they make in positioning themselves (Persson et al. 2020). Two conceptions of a good animal death at opposite sides of the spectrum will be sketched below.

### The pathocentric good death ideal

The pathocentric good death ideal represents the dominant view in the veterinarian profession, “deeply entrenched in the cultural narrative of pet ownership” (Pierce 2013, p. 477). The veterinary code of conduct by the Federation of Veterinarians of Europe (FVE) states that “[a]nimals should experience both a good life and a humane death without unnecessary suffering” (FVE 2019, p. 9). The guidelines on euthanasia of animals by the American Veterinary Medical Association (AVMA), which are frequently cited in the literature, go a step further in arguing that “[a] good death is tantamount to the humane termination of an animal’s life” (Leary et al. 2020, p. 6). It is to be done “with the highest degree of respect” (ibid. 13), which primarily seems to refer to the veterinarian’s duty to use those techniques that “induce the most rapid and painless and distress-free death possible” (ibid. 6). To prevent distress, euthanasia should ideally take place in a familiar, calm and safe environment, e.g., at home with the family holding and talking to their pet during the procedure. Anything that might induce negative emotions during euthanasia should be avoided.

Sociologist Patricia Morris talks of a whole dramaturgy that veterinarians engage in to create a good euthanasia experience for their patients as well as their clients:

From the veterinarian's perspective, the goal of a good client-witnessed euthanasia is a gentle slipping into death, which looks like an animal is quietly and painlessly falling asleep. […] Euthanasia is considered successful when the animal dies peacefully, the veterinarian maintains an appropriate presentation, and the owners are thought to have a good last memory of their pet. (Morris 2012, p. 76)

The AVMA guidelines stress the veterinarian’s and owner’s responsibility in determining the right point in time at which death will be good, i.e., a relief, for the animal:

Euthanasia as a matter of humane disposition occurs when death is a welcome event and continued existence is not an attractive option for the animal as perceived by the owner and veterinarian. When animals are plagued by disease that produces insurmountable suffering, it can be argued that continuing to live is worse for the animal than death or that the animal no longer has an interest in living. The humane disposition is to act for the sake of the animal or its interests, because the animal will not be harmed by the loss of life. Instead, there is consensus that the animal will be relieved of an unbearable burden. (Leary et al. 2020, p. 6)

At which point and on which basis an individual’s (future) suffering is deemed insurmountable is, of course, up for discussion and depends, among other things, on one’s own position concerning the harm of death and welfare theories (Persson et al. 2020). For the purpose of this paper, it suffices to know that the prevalent good death ideal in veterinary medicine indicates that the life of an animal can reach such a low quality that its termination becomes preferable. In fact, it is sometimes argued that pain and other negative mental states may be even worse for animals than for us, due to their inability to mentally transcend from the present and grasp the long-term benefits of a treatment. Animals *are* their pain, so to speak, and there is no way to make a dog comprehend that his intense suffering over the next couple of weeks will ultimately prolong his life (Rollin 2011).

Intentionally inflicting a lot of pain and distress on an animal must be well-considered, therefore. Especially during the dying process, suffering is to be avoided at all costs, extraordinary circumstances excluded. Euthanasia is thought of as a “compassionate treatment option” (Leary et al. 2020, p. 8f) and “ultimate kindness” (Hurn and Badman‐King 2019, p. 141), actively—and occasionally aggressively—promoted by veterinarians (Pierce 2013, p. 470; Andre 2003).

### The animal hospice good death ideal

The *animal hospice movement* originated in the USA in the 1990s and has since then begun to challenge the dominant view in the veterinary profession in two ways. Whereas the pathocentric good death ideal is primarily concerned with the termination and avoidance of pain, the animal hospice good death ideal, similar to its counterpart in human medicine, primarily aims to sustain or improve quality of life (Bishop et al. 2016; IAAHPC 2013).

Furthermore, the hospice good death ideal questions an automatic synonymization of good death with euthanasia and accepts “the pet owner’s ethical and legal right and responsibility to decide whether the terminally ill animal will die by euthanasia or by hospice-supported natural death” (Bishop et al. 2016, p. 343). Letting the animal die without euthanasia or palliative care, i.e., “[t]reatment that supports or improves the quality of life (QOL) for patients and caregivers by relieving suffering” (ibid. 342), is considered unethical and inhumane. If a client absolutely opposes to euthanasia for religious or other reasons, but the physical, social or emotional needs of the animal patient can no longer be addressed satisfactorily, palliative sedation combined with adequate analgesia is deemed an appropriate ethical alternative (ibid. 353).

In recent years, two different movements have developed within animal hospice. The dominant school of thought regards palliative treatment as a favored way to *postpone*, but not replace, euthanasia, whereas a smaller but increasingly influential school of thought pursues a hospice-assisted natural death by adequately treating “pain and other signs of discomfort under veterinary supervision until the natural death of the individual” (ibid. 342). The latter reveals a good death ideal that is closely oriented to the palliative approach in human medicine.

On their web page, the International Association of Animal Hospice and Palliative Care (IAAHPC) is careful to avoid an explicit positioning between the two schools, but also states, when talking in more general terms about hospice care:

Hospice recognizes dying as a normal process, whether or not resulting from disease, and sees the end of life as an opportunity for growth. Hospice exists in the belief that patients in the last phases of life deserve this care so that they might live as fully and comfortably as possible. Through appropriate care and the promotion of a caring community sensitive to their needs, patients and their families may be free to attain a degree of mental and spiritual preparation for death that is satisfactory to them. (IAAHPC 2013)

In a similar fashion, Jessica Pierce states:

Dying can be a time of opportunity. And for some people, the value of natural death is explicitly one of spiritual opportunity, as a time of transition from one kind of being to another. Although we cannot know what goes on inside the minds and hearts of our animals, perhaps we should be open to the possibility that they, too, might experience something profound as they die. (Pierce 2011b)

Consequently, some animal hospice proponents have expressed their displeasure with the fact that “[a]nimals frequently get euthanized because they are exhibiting normal signs of the dying process” (Bittel 2008a as cd. in Joswig 2014, p. 85). They criticize the human caregivers’ unfamiliarity with the process, resulting in an emotional overload as well as a misinterpretation of normal symptoms as indicators of suffering. Bishop et al. (2016, p. 353ff) state that even though there is limited empirical data on what animals experience during non-euthanasia-induced deaths, the knowledge gained from human palliative medicine suggests that hospice-supported natural death does not increase suffering. In fact, the authors tentatively seem to suggest that as about 50% of owners second-guess the morality of their decision to euthanize, an adequate and compassionate animal hospice care might help owners to overcome their pets’ deaths.

A concrete example for an anti-euthanasia position stems from Hurn and Badman-King (2019). In interweaving their observations as anthropologists at a multi-faith ashram in Wales with their personal experiences with their dying dog, they insinutated that a natural death might not only be in the owner’s, but also the animal’s best interest. The Skanda Vale Community in Wales radically opposes euthanasia of human as well as non-human beings, because its members consider it as a traumatic event that takes away the individual’s (and maybe the caregiver’s) dignity. Euthanasia is perceived as uncalm and overly hasty transition “from pain to just being dead” (Hurn and Badman‐King 2019, p. 150), and as a betrayal of the animal’s trust in its caregiver.

The community’s position certainly represents an extreme one, shaped by religious and spiritual beliefs. The authors warn, however, to dismiss it as irrelevant, because it raises important issues in the debate that are often neglected, such as whether a natural death may be ethically preferable to euthanasia from the perspective of the animal. Moreover, the position that an animal’s life is valuable in itself, independent of utilitarian analyses of future prospects, has gained attention. Some clients might want their pet to die naturally rather than to be euthanized for non-religious reasons as well, therefore (for an example, see Pierce 2011b).

These developments indicate a good death ideal within the animal hospice movement that is modelled very closely on the palliative good death ideal in human medicine, due to their similar understandings of non-hastened dying as a meaningful and possibly necessary element of a good death.

In sum, traditional views of what good death entails have been criticized in both professions. Whereas the palliative good death ideal in human medicine has been increasingly challenged by proponents of euthanasia, the animal hospice movement has begun to question the paradigm of the pathocentric good death ideal in veterinary medicine. Strikingly, these trends indicate a convergence, i.e., a movement towards one another, at first glance. Are these trends developing independently, or are they interdependent? We will tentatively argue for the latter. While the influence of human medicine on veterinary medicine is quite evident, the opposite is not as clear, however. Therefore, we will focus more on the possible ways how concepts of a good animal death might have found their way into human medicine.

## Influences of human medicine on good animal death ideals

Veterinarians frequently and explicitly refer to concepts, practices and techniques from human medicine. We will mention three aspects, where this influence with regard to good animal death ideals becomes particularly apparent: a growing emphasis on life-prolonging therapies and symptom management, the initiation of animal hospice, and, very recently, a debate on animal autonomy in the context of end-of-life decisions.

Already in 2011, Rollin asserted that veterinary clinics use “many of the most sophisticated diagnostic and treatment modalities found in human medicine” (Rollin 2011, p. 655). Ten years later, this includes CT scans, MRI diagnoses, dialysis, radiation treatment, chemotherapy and open-heart surgeries. Feline patients with kidney failure can even receive transplants in the USA, where the donor organ is typically obtained from a young (1–3 years old) shelter cat to be adopted by the client regardless of surgery outcome (Aronson 2011; AAHA 2020). Chemotherapy against lymphoma is currently under use in case of both human as well as canine patients with the purpose to extend lives.^10^ Owners of old or ill pets can fall back on items specifically designed for the needs of geriatric or chronically ill animals, e.g., dog carriers, car ramps or anti-slip rags, and use the services of mobile clinics or specialized dog sitters.

With respect to the second aspect, palliative care in human medicine looks back on several decades, so naturally, animal hospice pioneers are eager to learn from their colleagues’ knowledge and experiences. Concepts, procedures and techniques are explicitly being “borrowed” (Shearer 2019, p. 332), such as palliative sedation. Similarly to its counterpart in human palliative care, Shearer argues that sedation can be beneficial for animal patients which are unresponsive to palliative management and in a considerate state of distress while remaining conscious (ibid. 332f). Health care members from human medicine are explicitly invited to take part in this movement, illustrated, for instance, in Shearer’s wish for “collaboration between veterinary and human medicine” with the aim to “elevate the level of medicine for animal hospice patients” (ibid. 335).

Additionally, it seems that the experiences pet owners make with regard to human end-of-life care likely affect later decision-making for their terminally ill animals. Williams et al. (2017) found that when deciding for or against chemotherapy for their pets, 58% of owners reject it, largely due to their previous experience with chemotherapy for relatives and friends. Even though nearly three quarters also (wrongly!) assumed chemotherapy would extend the animal’s survival time for more than 1 year, the benefits of chemotherapy were not believed to counterbalance potential negative side effects. Statements included, e.g., “having seen a relative undergo chemo, I would be less inclined to agree to chemo for my dogs”, “after seeing what it does to a person, I am unsure as to do this to an animal that doesn’t understand” and “after seeing what it does to humans, I don’t think it is ethical” (Williams et al. 2017, p. 7).^11^ It is likely that this influence is in fact a mutual one because most people experience an animal’s death prior to that of a human being. Anecdotal evidence offered by veterinarians in case reports and in an interview study by the authors (unpublished) indeed suggests that owners routinely compare their prior experience of a beloved pet’s death with a close relative’s death and also make strong assumptions regarding good death ideals in human medicine (Rollin 1999; Tannenbaum 1995).

The third aspect we would like to draw attention to concerns autonomy or, to be more precise in this context, the ability and alleged right to determine one’s own death. At first glance, it seems that autonomy is a clearcut example of one of the main differences between human and animal patients. Most adult humans can give their informed consent in being killed, whereas animals cannot. In accordance with that, references to end-of-life situations in veterinary medicine by advocates of (voluntary) euthanasia typically serve the purpose not to allude to the similarities between human and animal dying but to the dissimilarities. The end-of-life situations of (most) animals and humans are in fact *not* being perceived as analogous, consequently. Instead, the reference to the way how we treat our companion animals at the end of their lives serves to illustrate a blatant injustice – whereas ‘even a dog ‘, i.e., a being that cannot express its consent to being killed, is euthanized, an autonomous human patient, who repeatedly and comprehensibly expresses her rational wish to be killed at her own request, is not. Along this line philosopher John Shand argues that “[o]ur moral justification for being allowed to end the lives of sick animals might be considered weakened precisely because they cannot grant their reasoned consent to it, whereas humans can consent to euthanasia” (Shand 2018).

This being said, there are also intentions to adapt the concept of autonomy to animal patients on the grounds that “animals share with humans the decisional and volitional capacities that underlie autonomy and agency “ (Pierce 2019, p. 426). In a similar fashion, Grimm and Huth (2016) speak of the owner’s and veterinarian’s duties to meet the animal’s (presumed) will in end-of-life decision-making. Pierce (2019) illustrates what this duty might actually entail by help of the example of a voluntary stop of eating and drinking. Just as it is taken as a decision against continuing to live in human patients (after it has been ruled out that the inappetence is not caused by underlying medical problems), it should be equally respected in case of animals, according to the author.

In human medicine, great endeavors have been made to extend the principle of autonomy to as many patients as possible, e.g., children, mentally impaired or demented patients. We tentatively raise the possibility that the above authors implicitly refer to these trends, further diminishing the gap between human and animal patients. To our knowledge, this alleged mutual influence between end-of-life experiences in human and veterinary medicine has not yet been investigated on a large scale, however. Additionally, there has been little discussion on the moral justifiability of using chemotherapy, palliative sedation and other advanced medical therapies for animal patients.

## (Possible) influences of veterinary medicine on good human death ideals

In contrast to the situation in veterinary medicine, palliative care providers scarcely refer to concepts, practices or techniques from veterinary medicine. Consequently, potential influences will be rather implicit. Moreover, ideas of good death and dying vary more with regard to humans than they do for companion animals, because human medicine deals with very different kinds of patients; from newborns, children and autonomous adults to comatose or mentally impaired adults or patients suffering from advanced dementia. This makes it harder to pinpoint unambiguous examples of interferences. Interferences are most likely to be found with regard to non-autonomous patients, who are unable to give their informed consent, e.g., infants or cognitively impaired patients. It is with regard to these patients that end-of-life situations between the two professions are the most comparable. We will get into further detail of two aspects indicating this influence with regard to good human death ideals. The first refers to alleged fundamental differences between human and animal dying, the second to the killing of animals and humans.

### Dying humans and dying animals

Veterinary medicine has traditionally acted on the assumption of a pathocentric good death ideal, as we have seen. In a nutshell, it states that when an animal has reached or is expected to reach a level of insurmountable suffering, euthanasia is regarded as a relief of an unbearable burden (Leary et al. 2020, p. 6). The EAPC, in contrast, supports a non-induced death. However, it is not self-explanatory why an abbreviation of the dying process should not be considered as preferable. If it is assumed that non-hastened dying is not merely considered to be in the family’s or the health care team’s but first and foremost the *patient’s* interest, then there must be other reasons besides unspecific references to the naturalness of dying or the worthiness of life that make a non-induced death preferable from this point of view.

One such a reason would be the belief that dying has transformative potential right until the end. Eliminating this meaningful experience, e.g., by preponing the time of death, would then necessarily lead to a comparatively worse death. In line with these considerations, the claim has been defended that euthanasia can be the morally right thing to do in case of animals, but never when it comes to humans.

The pet’s suffering is gross because the brute animal naturally cannot rise above and reflect upon what it bears. Instead, we find the pet entirely immersed in its pain and afflictions. Accordingly, the pet has nothing to gain, no new insights to derive from experiencing its own mortality. In fact, it does not experience its own mortality; it simply labors under a burden. Unlike the pet, we understand this burden and its name: mortality. [...] The animal, however, remains dumb and mute even at the end of its life, a terminus that it neither comprehends nor contemplates. Indeed, partially for this reason, it is pathetic. It merits pity. No good can come of the animal’s endurance of its ending. In light of this we humanely kill a pet whose life holds no prospects for growth as it ends. (Cavanaugh 2016, p. 18)

It is crucial to note that this difference between human and animal dying is thought to constitute a *fundamental* difference, independent of individual abilities and deficits (see also Bachelard 2002). As a consequence, it is not admissible to extrapolate from justified animal euthanasia to the rightfulness of euthanizing humans. Euthanasia is described as trivializing human death and even “do[ing] dirt on human life “ (Cavanaugh 2016, p. 28, see also Anscombe 2005) by falsely equating a human being with a pet. The request for euthanasia thus does not merely represent an ill-guided good death ideal, but a failure to acknowledge one’s human nature.

Such a position can be criticized, however. One could argue that the opportunities that death ‘promises’ are for each individual to choose and, crucially, might not even be applicable to many patients. But if a patient is either unwilling to submit to ‘natural’ dying as a transformative experience or unable to contemplate her mortality and comprehend her impending death—what is it that makes this dying still so fundamentally different from an animal’s? In accordance with Cottrell and Duggleby’s (2016) assertion that society is under change to become a dying-denying society, some people may no longer share the view that a non-induced dying is always ‘good‘. A slow, non-hastened and potentially painful or frightening death would then be represented as a needless evil, which can be discarded by means of euthanasia. So why, one might ask, should I be forced to endure the whole messy process, while my dog is mercifully and quickly put to sleep?

Reflections of this kind are frequent, put forward by all kinds of writers in newspapers and magazines, blogs, interviews, etc. (see, e.g., Baggini 2015; Baume 2009; Pierce 2011a; Pierre 2018; Shand 2018). They thus have already found their way into hospitals, hospices and nursing homes, where they potentially clash with the good death ideals held by palliative physicians and caregivers. This bears the potential for deep conflicts.

Simultaneously, some animal hospice advocates argue for the potentially transformative character of death also in terms of animals, as we have seen. They represent an extremely minor position until now, but objections to euthanasia could be expected to become more frequent, especially if pet owners had positive experiences with palliative human medicine in the past.

### Killing humans and killing animals

It has become evident that some authors consider human dying to be fundamentally different from animal dying. This is closely related, but not reducible, to the claim that there is a morally justified taboo of killing fellow humans but not animals. Whereas animals can (or maybe even should) be euthanized at the end of their lives, the same does not apply to humans. This view, also known as the *sanctity of life position*, has been rejected by several authors, however (e.g., Singer 2011; Kuhse 1987). Here, we would like to focus on two authors, who very recently challenged the sanctity of life view with explicit references to euthanasia of both animals and humans.

These authors do not deny that we are more reluctant to kill a member of our own species than another, but seek to find ethically neutral explanations. Joseph Pierre, a psychiatrist who wrote a newspaper article about his dying dog, suggests that our different attitudes regarding the euthanasia of humans in contrast to animals have multiple sources. First of all, the killing of animals for all kinds of purposes has been a significant element of human everyday life since the beginning of humankind. This divide between humans and animals was even further cemented with the rise of Judeo-Christian and Islamic religions, according to the author, because both strongly oppose to the killing of humans but at the same time question the existence of animal souls (Pierre 2018).

As religion has been losing its influence in many Western civilizations, it is plausible to assume that “the historical divide between our attitudes towards euthanasia for humans and animals is narrowing” (ibid.). The acceptance of euthanasia rises among the non-professional population, but at the same time, our deep-rooted inhibition against killing a member of our own species, persists. As Pierre describes it: “Although ‘death with dignity’ is increasingly supported in many parts of the world, often neither doctors nor patients seeking death want to ‘push the plunger’ and take responsibility for being the hand of death. In this sense, euthanasia remains a hot-potato issue in human medicine” (ibid.).

Pierre does not explicitly say so, but he seems to think that passive euthanasia and palliative sedation merely conceal the fact that we are not brave enough to give people the best death possible. This becomes even clearer in light of the fact that he asked to take an active part in the euthanasia of his dog by assisting the veterinarian in administering the lethal injection. For Pierre, the right thing to do was to overcome his strong reluctance in killing his dog.

Frans Stafleu, a Dutch veterinarian with a research focus in animal ethics, argues along a similar line, but comes to slightly different conclusions. Just like Pierre, he is convinced that our reluctance to kill another being is higher if the said individual is a fellow human being. Stafleu also believes that this taboo sometimes has ethical significance, if only a very marginal one. To demonstrate this, Stafleu introduces two hypothetical cases: an 89-year-old Alzheimer's patient and an old dog with breast cancer and arthrosis. Both patients suffer from pain and other negative symptoms and their cognitive abilities are on a comparable level, according to the author. The Alzheimer's patient has lost her ability to make autonomous choices and can no longer give her consent to medical interventions (Stafleu 2016, p. 104).

The respective doctors and family members agree that death would be preferable to ongoing suffering for both individuals by expressing their wish that death will soon occur. So why is it, Stafleu asks, that in case of the dog, we actively *initiate* death, whereas in case of the human patient, we merely *hope* for a merciful death in the form of a pneumonia?

Stafleu, arguing from a distinctive utilitarian standpoint, discusses and dismisses several options that might be said to constitute a morally relevant difference and concludes that the two end-of-life situations are indeed analogous. Consequently, they should be treated similarly as well. As Stafleu considers euthanasia to be almost self-evidently preferred over natural death, this means that both patients should be ‘put to sleep’.

This being said, Stafleu also draws attention to one major difference between the two individuals in his example, which he suggests to be (marginally) morally relevant insofar that it permits us to *delay* euthanasia in case of the Alzheimer's patient but not the dog. Whereas we are obliged to not let suffering become intense in case of the animal, we are justified in waiting a bit longer than would be in the patient’s actual best interest in case of the old woman. Stafleu justifies this claim as follows:

[H]umans normally have a concept of death and the wish not to die. Although our patient does not have these capacities any more, we still see the echo of the past here, for we still recognize our mother, Mrs Janssen etc. in this patient. We tend to ask ourselves what she would prefer in this situation. If there is no clear answer (like in our case) we feel uneasy, because we place a lot of weight on the autonomous opinion of fellow humans. The fact that we lack such a vital element for decision making certainly adds to the already existential moral uncertainty. (ibid. 111)

For Stafleu, this feeling of uncertainty justifies a more cautious procedure than in the case of the dog, but it is not strong enough to outweigh the patient’s best interest. Actively killing the Alzheimer's patient in this scenario thus “is justified and (perhaps) even a moral obligation” (ibid. 112).

Opponents of the sanctity of life view typically also argue for the irrelevance of species membership: that one patient is a human being and the other one is a dog is not in itself ethically relevant.^12^ It is noteworthy that arguing in favor of the ethical irrelevance of species membership is not in conflict with the position that there is a major difference between *most* humans and *most* animals, attributable to, e.g., certain cognitive capabilities. Consequently, one could hold that autonomy, e.g., is of major ethical significance for end-of-life decisions. As Stafleu also defines autonomy as a cognitive capability that can dissolve in the course of certain neurodegenerative diseases, however, the Alzheimer's patient from the example above can be regarded as having lost what once separated her from animals in a morally relevant way.

Patients with a similar view might therefore feel that certain diseases ‘reduce’ them to their animal nature, so to speak. The wish to be treated like a dog at the end of one’s life could, in conclusion, reflect the belief of becoming morally relevantly similar to the dog. The ability to reflect one’s mortality or to ‘grow’ through death, which the patient might have judged to be extremely significant before, would become pointless. In fact, the palliative good death ideal, with its emphasis on self-reflection and insight, may not be achievable for a growing number of patients to begin with (see also Steffen-Bürgi 2009). Even if a good palliative death might be interpreted to be off the table for these patients, a good animal death still represents an option.

The debate around the ethical (ir)relevance of species membership has been going on for several decades now. We will not attempt to solve it here, but instead caution that it can and should not be dismissed lightly. To begin with, both human as well as veterinary medical ethicists would be well-advised to engage in a thorough analysis of the dissimilarities but also similarities of their patients and their respective experiences at the end of their lives.

## Discussion

To our knowledge, the interferences between human and animal death ideals have not been normatively reflected, yet alone registered by palliative physicians opposing euthanasia. Veterinarians, in contrast, have repeatedly done so, arguing, for instance, that “health care providers and those involved in creating legislation would do well to seek out and embrace this wealth of veterinary experience with assisted death “ (Rothenburger 2015, p. 624). These remarks can both be read as a criticism against the rising animal hospice movement (explicitly modeling itself on its equivalent in human medicine) as well as a clear positioning in favor of euthanasia for human patients.

A particularly self-confident example is brought forward by Ruth Eyre-Pugh and James Yeates, who express their “concern […] for the welfare of animals, near the end of their lives, whose owners are led to believe by the current situation in human medicine in the UK that dying naturally is normal and a certain amount of suffering is acceptable “ (Eyre-Pugh and Yeates 2017, p. 10). To make this point stronger, the authors quote another statement from a colleague, who described the following incident in an online forum for veterinary surgeons:

Modern medicine has made protracted death far more likely than before, and I think we vets rightly become accustomed to only seeing and expecting a swift and painless end. A GP friend called me recently to discuss euthanasia of their dying old dog and so I packed my bag to go. However, a few minutes later she called back to say that she and her husband (also a medic), had decided to allow 'things to happen naturally'. And so they did, and it took a couple of days... I was mortified, but then realised that this was what they were used to seeing and they saw nothing wrong. (Eyre-Pugh and Yeates 2017, p. 10)

The natural death movement with its emphasis of a non-hastened dying process is in fact strongly influenced by the palliative good death ideal, as has become evident. For Rollin, Eyre-Pugh and Yeates as well as other veterinarians, this development is alarming. In fact, they believe that “due to their training and broad experience veterinarians appear to take a more holistic approach to end of life issues than doctors, in particular with regard to assessing quality of life “ (Eyre-Pugh and Yeates 2017, p. 11). Veterinarians are explicitly encouraged to “share their expertise in the debate about euthanasia for humans” (ibid.).

Current developments within the veterinary profession and the status of pets as family members entail the danger of unqualified anthropomorphization and, thus, unjustified treatment. The convergence of veterinary and human medical technology, treatment options and quality of life assessment tools can occasionally give the impression that two medical cases in human and veterinary medicine are similar and should be treated similarly as well, but the opposite may be the case. Animals cannot give their consent in a chemotherapy or palliative sedation, nor can we make them understand that a therapy represents a necessary evil but will turn out to be beneficial in the long run. Even in human medicine, it makes a crucial difference whether the patient in question is herself able to give her consent or whether others are obliged to make a substitute-decision. Autonomy plays an important role for end-of-life situations in human medicine. The situation in the veterinary clinic may at best be comparable with pediatric or cognitively severely impaired patients, thus. Veterinarians may be well-advised to shift their focus from standard palliative care to end-of-life decision-making in pediatrics, geriatrics or other better-suited areas. Against this background it is worrying that pet owners may be affected by past experiences with human palliative care when making a decision for their terminally ill pet.

To sum up, veterinarian palliative medicine is confronted with the problem that concepts and procedures from human medicine have already gained access into the profession, whereas a thorough analysis of whether and to what extent they *can* or *should* be transferred has been neglected in the discussion. Clearly, the techniques of palliative sedation or chemotherapy *can* be applied to animal patients—but whether this *ought* to be done in consideration of the patient’s interests is a different question.

Veterinarian and palliative care specialist Robin Downing cautioned that there is a tendency within the animal hospice movement of “translating and applying the exact same principles of human hospice to our animals, including an absolute prohibition against euthanasia “ (Pierce 2011b). Already in 2011, Downing feared that the pendulum, to adopt the author’s figure of speech, might swing from a pathocentric good death ideal with its overly positive view of euthanasia as effective treatment of pain to an anti-euthanasia position in the future. Lisa Moses admonishes that veterinarians “have barely begun to have widespread public discussions about what end-of-life care for animals should ideally be and whether we want to avoid the pitfalls of human health care at the end of life” (Moses 2019, p. 389).

In addition, animal hospice—like veterinary medicine in general—attaches great importance to the family’s perspective, much more, in fact, than is the case in human medicine. As has been seen above, religious or spiritual beliefs, preferences or values of the animal’s owner with regard to the good death ideal are to be respected as far as possible. Thus, a veterinarian is to accept a client’s rejection of euthanasia and do her best to fulfill expectations.

Rollin admonishes that because “trying everything [to keep the patient alive for as long as possible] is what veterinary specialists *do*” (Rollin 2011, p. 655), the ongoing specialization of veterinary medicine increases the danger of overtreatment in end-of-life care and might result in a reluctance on behalf of the client and the specialist to let go. In fact, he believes that the rise of advanced high-quality veterinary medicine and specialization has led to a convergence of veterinary and human end-of-life care in the wrong direction:

In a major ironic twist of fate, rather than human medicine learning the wisdom of euthanasia for suffering animals from veterinary medicine, in the ensuing 30 years veterinary medicine assimilated some of the more pernicious aspects of human medicine. In particular, as animals became increasingly viewed as ‘members of the family’ the reluctance to euthanize began to enter veterinary medicine. (ibid. 653)

Without engaging in systematic discussions on the similarities and differences between all different kinds of human and non-human patients, the claim that human medicine should move towards veterinary medicine due to the latter’s greater expertise or a supposedly more holistic approach, becomes extremely problematic, however. Instead of assuming that there can only be one ethically right direction of convergence, veterinarians are advised to engage in a conversation with those palliative physicians opposing euthanasia and PAS first, in order to understand their reluctance and worries.

In human medicine, patients are, on the one hand, encouraged to use advance care directives to clearly indicate their preferences regarding, e.g., life-prolonging treatments, with the promise that these preferences are to be respected by the health care professionals. On the other hand, the widespread wish to be painlessly killed at a self-given point in time cannot be fulfilled, as euthanasia or PAS is still illegal in most countries worldwide (Cohen et al. 2013). Palliative medicine attempts to release this tension by presenting the patient with alternatives to euthanasia, which strives to minimize suffering and distress right until death, to the point of palliative sedation (see, e.g., Lima et al. 2016; Radbruch et al. 2016).

Of course, it would be absurd to claim that these developments result (only) from veterinarian practices. They might be read, however, as concessions to those for whom a good death has much more similarity with what is typically being considered a good companion animal death and who reject the sanctity of life position. For future research, it would be interesting to know if pet owners tend to have similar good death ideals for themselves as for their pets, or whether these are distinctive. In either case, palliative physicians are well advised not to dismiss a patient’s request for euthanasia as ill-guided or a wrong assessment of quality of life. Instead, they should be open to the possibility that their patients’ notions of a good death might occasionally differ from their own. How we want to handle these diverse good death ideals on a societal level is subject to yet another discussion.

## Conclusion

In human medicine and, to a smaller extent, in veterinary medicine, ideals of a good death are becoming more diverse. In human medicine, the growing diversity is mainly due to the plurality of preferences of the patients themselves, whereas in veterinary medicine it seems to be based, first and foremost, on an acceptance of the clients’ versatile good death ideals rather than a scientifically based new understanding of what might be in the animal patients’ best interests. Despite good intentions, a poorly reflected adaptation of concepts and procedures from human medicine could severely damage the animal patients’ wellbeing. Vice versa, ignoring the debate on the (dis)analogies of human and animal dying and end-of-life decision-making risks to disregard the fact that considerations of a good human death can considerably vary. Palliative care providers and medical ethicists are thus invited to engage in the debate on whether human and animal dying actually represent two fundamentally different kinds, if that difference applies to all human patients and if so, on what grounds. The same applies to criticism on the sanctity of life view that has been brought forward by philosophers and euthanasia advocates.

Regardless of whether the convergence between human and animal good death ideals is welcomed or not, veterinary as well as human medicine would greatly benefit from a shared discourse, where similarities as well as dissimilarities of their various patients and individual end-of-life situations are being carved out and, in a further step, normatively reflected. It is only then that postulations and requests on the part of patients, relatives, veterinarians or right-to-die organizations, e.g., with regard to the alleged (dis)analogy between human and animal dying or killing, can be adequately met.

## Footnotes

1 The terms *pet*, *companion animal* and *animal* will be used interchangeably throughout this article, unless explicitly indicated otherwise. Animals in the focus of our interest will be those sharing a close emotional relationship with their owners, therefore, such as dogs and cats.

2 It may be argued that species membership constitutes a morally relevant difference, making efforts to compare the good death ideals of human with veterinary medicine obsolete or at least uninteresting due to the lack of practical implications. We do not take this to be true for at least two reasons. For one thing, this issue remains one of the most heatedly debated in philosophy. The possibility that some human and animal end-of-life situations may in fact have equal ethical implications due to their morally relevant similarities should not be dismissed lightly, therefore. Furthermore, even if someone is personally convinced that there are morally relevant dissimilarities between *all* animals and *all* human beings, a descriptive analysis of how corresponding good death ideals or end-of-life situations change and influence one another, is important in its own. Modern palliative medicine acknowledges a patient’s right of self-determination as well as individual values and worldviews. It should be prepared to being frequently confronted with quite different views on these matters, therefore. We will get into more detail on two aspects where species membership could and has been argued to (not) constitute a morally relevant difference below.

3 When referring to *dying* or the *dying process*, we will typically have in mind the last days or hours in the life of an individual, unless indicated differently. Our focus in this paper is not so much dying as a preparedness to one’s death or the acceptance of one’s mortality—even though these points will be mentioned and discussed throughout the paper—but dying understood as the very last phase of life. *Death* will be understood in a comprehensive way. A *good death* encompasses many more aspects in our understanding, thus; including, but not limited to, the very last moments in an individual’s life.

4 In fact, Radbruch et al. acknowledge that due to incompatible normative frameworks among European palliative care providers, full consensus on these matters will be unachievable. For instance, there is considerable dissent among the members of the EAPC on whether the provision of euthanasia and physician-assisted suicide should be integrated into the practice of palliative care, or whether they constitute opposites (see Radbruch et al. 2016, supplemental material, Table 1). For this reason, the palliative good death ideal that is being described here and hereafter should not be mistaken as a consensual position among European palliative care providers, but as one (albeit prominent) position.

5 This paper is concerned with euthanasia, but most deliberations also apply to physician assisted suicide (PAS). As our focus here lies on the comparison to veterinary medicine, we will not go into detail of morally relevant similarities and dissimilarities between euthanasia and PAS.

6 Radbruch et al. (2016) emphasize that wishes to die should not be taken at face value as they are complex, often ambivalent and require a careful and sensitive communication with the patient. This issue is of high ethical and practical relevance, but what we are interested in here is what this passage tells us with regard to more general views on the patient’s quality of life within the palliative good death ideal.

7 Whenever we will speak of a ‘natural’ death hereafter, we will refer to this understanding. ‘Natural’ death thus will be used as a shorthand for a non-hastened, non-prolonged and non-induced dying process. It does not refer to the withholding of pharmaceuticals, symptom management, medical technology or the like.

8 The article is no longer included in more recent editions.

9 The terms *pet owner*, *caregiver* and *client* will be used interchangeably, as they typically refer to one and the same person.

10 Untreated dogs have an average survival time of about one month, whereas chemotherapy leads to a life prolongation ranging from 2 to 24 months (Stephens 2019, p. 2).

11 It is interesting to note that these statements also reveal an uncertainty about whether both situations can and should be regarded as analogous or not.

12 The view of the moral irrelevance of species membership does not necessarily lead to a pro-euthanasia attitude. Jessica Pierce takes an assimilationist position on the matter, challenging not only our reluctance in euthanizing humans but also our *lack* thereof when it comes to killing animals. According to her, we should probably be more open to euthanasia in case of humans and more reluctant in case of animals.
